# Auxin-Dependent Cell Elongation During the Shade Avoidance Response

**DOI:** 10.3389/fpls.2019.00914

**Published:** 2019-07-12

**Authors:** Lin Ma, Gang Li

**Affiliations:** ^1^College of Life Science and Technology, Jinan University, Jinan, China; ^2^State Key Laboratory of Crop Biology, College of Life Sciences, Shandong Agricultural University, Tai’an, China

**Keywords:** auxin, cell elongation, shade avoidance response, photoreceptor, PIFs

## Abstract

Plant uses multiple photoreceptors and downstream components to rapidly respond to dynamic changes in environmental light. Under shade conditions, many species exhibit shade avoidance responses that promote stem and petiole elongation, thus helping plants reach the sunlight. In the last few years, the regulatory molecular mechanisms by which plants respond to shade signals have been intensively studied. This review discusses the regulatory mechanisms underlying auxin-mediated cell elongation in the shade avoidance responses. In the early response to shade signals, auxin biosynthesis, transport, and sensitivity are all rapidly activated, thus promoting cell elongation of the hypocotyls and other organs. Under prolonged shade, increased auxin sensitivity—rather than increased auxin biosynthesis—plays a major role in cell elongation. In addition, we discuss the interaction network of photoreceptors and Phytochrome-Interacting Factors, and the antagonistic regulation of Auxin/Indole Acetic Acid proteins by auxin and light. This review provides perspectives to reframe how we think about shade responses in the natural environment.

## Introduction

Light is a vital resource for green plants, as it provides an energy source for photosynthesis and acts as a signal to direct plant growth and development. In leaves at the top of the canopy, chlorophylls and other pigments specifically absorb blue (400–500 nm, B) and red (600–700 nm; R) wavelengths of light. By contrast, plant tissues transmit or reflect most far-red (700–750 nm; FR) wavelengths of light, leading to strong enrichment of FR light under the canopy. Under canopy shade, the intensity of B and R light, as well as Photosynthetically Active Radiation (400–700 nm; PAR) are reduced, whereas green and FR light intensities are relatively enriched; these dynamic changes in light quality and intensity trigger specific shade responses (Sellaro et al., 2010; Casal, 2013; Pierik and de Wit, 2014; Pedmale et al., 2016; Ballare and Pierik, 2017; Fiorucci and Fankhauser, 2017). Under shade conditions, most shade-intolerant species (e.g., *Arabidopsis thaliana*) exhibit shade avoidance responses, including enhanced cell elongation in various organs (hypocotyl, petioles, internode, stem, and branches), increased hyponastic growth of leaves, and accelerated flowering time (Franklin, 2008; Casal, 2012, 2013). When growing taller is not an option, some plants exhibit a series of tolerance-related morphological and physiological changes, including expanded leaf size and area, decreased leaf thickness, and reduced chlorophyll a:b ratio, thus increasing plant performance and the efficiency of light capture under shade and dim-light conditions (Gommers et al., 2013). In this review, we focus on the current understanding of auxin-mediated cell elongation under various shade conditions in *Arabidopsis thaliana*.

### Responses to Low R:FR Shade Signals

In Arabidopsis, the photoreceptor phytochrome A (phyA) mediates the response to continuous FR light and phyB mediates the response to continuous R light (Li et al., 2011; Wang and Wang, 2015). The disruption of *phyB* leads to constitutive shade avoidance responses, consistent with its negative role in this response (Reed et al., 1993). Arabidopsis PHYTOCHROME-INTERACTING FACTORS (PIFs, including PIF3, 4, 5, and 7), a subfamily of basic helix-loop-helix (bHLH)-type transcription factors, physically interact with phyB and positively regulate the shade avoidance response by directly inducing the transcription of growth-promoting genes (Leivar et al., 2012a,b; Li et al., 2012). Under low R:FR conditions, PIF3 has a minor role in the shade avoidance response, PIF4 and PIF5 play redundant roles, and PIF7 plays a more prominent role in mediating shade-induced cell elongation (Hornitschek et al., 2012; Leivar et al., 2012a,b; Li et al., 2012; de Wit et al., 2015). The transcription of genes encoding other bHLH, or HLH-type transcriptional regulators, including LONG HYPOCOTYL IN FAR-RED1/SLENDER IN CANOPY SHADE1 (HFR1/SICS1), PHYTOCHROME RAPIDLY REGULATED1 (PAR1), PAR2, AND PIF3-LIKE1 (PIL1), is also rapidly induced by low R:FR shade signal. These factors negatively regulate shade avoidance responses through physically interactions with PIF4 and PIF5, forming non-DNA binding heterodimers and inhibiting transcriptional activation of PIF4 and PIF5 downstream targets (Salter et al., 2003; Roig-Villanova et al., 2007; Hornitschek et al., 2009). In addition, other well-studied components of the light signaling pathway, including the basic leucine zipper (bZIP) transcription factor ELONGATED HYPOCOTYL5 (HY5), B-Box (BBX) transcription factors (including BBX21, 24, and 25), and the E3 ligase CONSTITUTIVELY PHOTOMORPHOGENIC1 (COP1), are involved in shade responses (Crocco et al., 2010; Sellaro et al., 2011; Rolauffs et al., 2012; Pacin et al., 2016; van Gelderen et al., 2018; Ortiz-Alcaide et al., 2019).

When plants perceive low R:FR, the expression levels of thousands of genes are rapidly altered, thus allowing plants to respond to shade conditions. A recent meta-analysis of public transcriptome data of shade responses identified a set of core response genes, including 98 up-regulated and 112 down-regulated genes (Sellaro et al., 2017). These core response genes include well-known markers of the shade avoidance response, such as *HOMEOBOX*2 (*HB2*), *HFR1*, and *IAA29*. Interestingly, a large proportion of core shade-upregulated genes are the direct targets of PIF3 (∼40%), PIF4 (∼80%), PIF5 (∼50%), and AUXIN RESPONSE FACTOR6 (ARF6; ∼60%), further confirming that PIFs and ARFs play critical roles in shade responses (Sellaro et al., 2017).

### Auxin Biosynthesis and Transport Are Induced During the Early Shade Response

#### Shade Signal Perception

**FIGURE 1:**
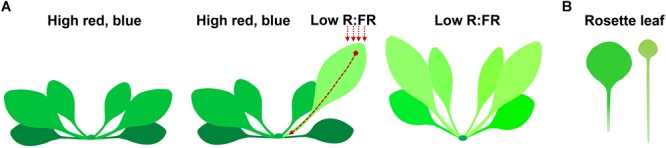
Morphological changes in plants under shade conditions. **(A)** Low R:FR shade treatment of a single leaf (middle) and whole plant (right) induces leaf hyponasty compared to the unshaded control plant (left). Arrows indicate the position of low R:FR shade treatment, which induces auxin biosynthesis at the site of shade signal perception. The newly synthesized auxin is transported out to the petiole, inducing cell elongation of petiole and hyponastic growth of leaf. **(B)** Prolonged shade treatment increases petiole elongation and reduces leaf size (right, compare to the unshaded control on the left).

In Arabidopsis, rosette leaves and cotyledons are the major sites of low R:FR shade signal perception. Within 1 h of low R:FR treatment, free indole-3-acetic acid (IAA) contents in Arabidopsis shoots increased by over 50% (Tao et al., 2008; Li et al., 2012; Kohnen et al., 2016). This newly synthesized auxin is subsequently transported out to the bases of the lamina, petiole, and hypocotyl, where locally synthesized and newly transported auxin promote cell elongation in the petiole and hypocotyl (Casal, 2013; Kohnen et al., 2016; Michaud et al., 2017; Pantazopoulou et al., 2017; Iglesias et al., 2018). Subjecting the tip of the cotyledon or leaf to low R:FR significantly induces hyponastic growth. Interestingly, this only occurs in the abaxial side of the petiole of the leaf that has perceived FR, but not in other rosette leaves (Figure 1) (Muller-Moule et al., 2016; Michaud et al., 2017; Pantazopoulou et al., 2017; Kim et al., 2018). By contrast, subjecting whole plants to low R:FR conditions induces elongation of both the abaxial and adaxial sides of the petiole (Figure 1; Michaud et al., 2017; Pantazopoulou et al., 2017).

#### Auxin Biosynthesis and Metabolism

In Arabidopsis, auxin biosynthesis starts with the conversion of tryptophan (Trp) to indole-3-pyruvic acid (IPA), catalyzed by TRYPTOPHAN AMINOTRANSFERASE OF ARABIDOPSIS1 (TAA1), which is encoded by *SAV3* (*SHADE AVOIDANCE3*) (Tao et al., 2008). In turn, IPA is converted to IAA by the YUCCA (YUC) family of flavin monooxygenases (Won et al., 2011; Zhao, 2018). The *sav3/taa1* and *yuc2,5,8,9* quadruple mutants show reduced auxin accumulation and short hypocotyls under low R:FR conditions, indicating that TAA1, YUC2, 5, 8, and 9 act additively and are required for shade-induced auxin biosynthesis (Tao et al., 2008; Kohnen et al., 2016; Muller-Moule et al., 2016). Although TAA1 is required for low R:FR-induced auxin production, *SAV3*/*TAA1* transcription is not directly induced by shade (Tao et al., 2008). In contrast to *SAV3*/*TAA1*, the transcription of *YUC2, 5, 8*, and *9* is rapidly and strongly induced within 1–2 h, or even 15 min, of low R:FR treatment (Tao et al., 2008; Li et al., 2012; de Wit et al., 2015; Kohnen et al., 2016; Muller-Moule et al., 2016). *YUC2* and *YUC5* are strongly expressed in the vascular tissues of petioles and hypocotyls, whereas *YUC8* and *YUC9* are strongly expressed in mesophyll and vascular cells of the leaf margin but not in hypocotyls (Challa et al., 2016; Muller-Moule et al., 2016). *YUC3* is also significantly induced in hypocotyl after 45 min low R:FR treatment and is required for shade-induced hypocotyl elongation (Kohnen et al., 2016).

In addition to *SAV3*/*TAA1* and *YUCs*, auxin homeostasis-related genes are also involved in shade responses. Disruption of *REVERSAL OF SAV3* (*VAS1*) not only rescued the impaired avoidance response of the *sav3/taa1* mutant under low R:FR conditions, but the *vas1 sav3* plants also exhibited a mild constitutive shade response phenotype even under high R:FR conditions, indicating that VAS1 negatively regulates shade avoidance responses (Zheng et al., 2013). Biochemical analyses revealed that VAS1 and TAA1 have opposing biochemical functions; therefore, VAS1 inhibits shade response by inhibiting auxin production (Zheng et al., 2013).

**FIGURE 2:**
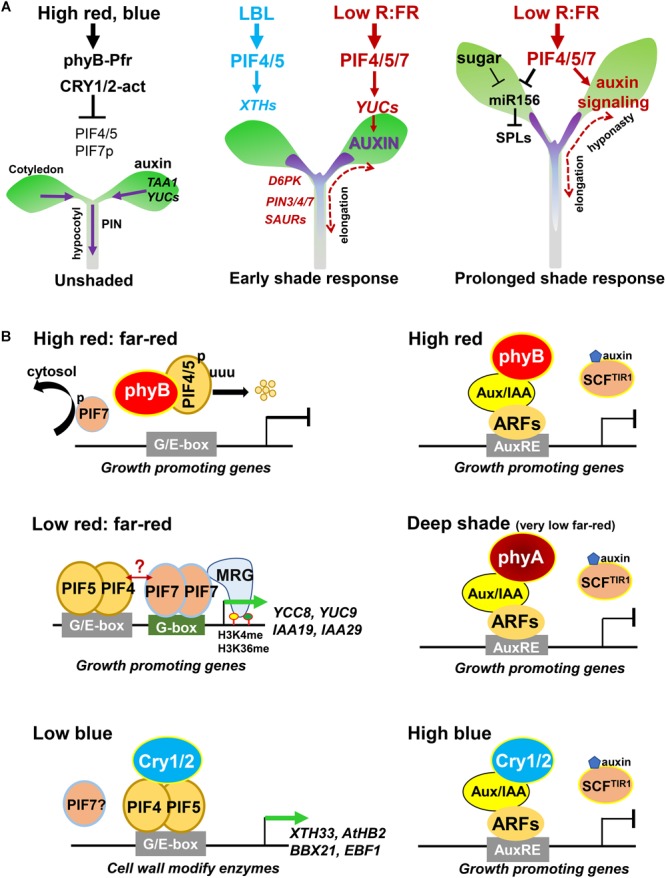
Regulatory mechanisms of the shade response in *Arabidopsis thaliana*. **(A)** The effects of light on hypocotyl elongation. Left, high-intensity red and blue light inhibits the functions of PIF4/5/7 by increasing the activity of phyB and CRYs under unshaded conditions. Middle, during the early shade response, LBL regulates the expression of genes encoding cell wall-modify enzymes through PIF4/5; low R:FR signal activates PIF4/5/7, thus promoting auxin biosynthesis in the cotyledon, which is then transported out to the hypocotyl where it induces cell elongation. Right, PIF4/5/7 increase auxin sensitivity and inhibit the expression of *miR156* to mediate the prolonged shade avoidance response. **(B)** Physical interactions of different photoreceptors, PIFs, Aux/IAA, and ARF proteins under different light conditions.

VAS2/GH3.17 (GRETCHEN HAGEN3.17) catalyzes the conjugation of free IAA to inactive IAA-Glu (IAA-glutamate). Disruption of *VAS2/GH3.17* resulted in accumulation of free IAA, thus enhancing shade-induced hypocotyl elongation (Zheng et al., 2016). Interestingly, low R:FR treatment suppresses the transcription of *VAS2/GH3.17* and its two homologs, *GH3.18* and *GH3.19*, suggesting that shade induces the accumulation of free IAA not only by promoting its synthesis and transport in the cotyledon, but also by reducing the conjugation of free IAA in the hypocotyl (Salter et al., 2003; Zheng et al., 2016). A recent study revealed that GH3 family protein FAR-RED INSENSITIVE 219/JASMONATE RESISTANCE1 (FIN219/JAR1/GH3.11), negatively regulates shade avoidance responses by modulating auxin homeostasis (Swain et al., 2017). These findings confirm that local auxin metabolism in the hypocotyl plays critical roles in the shade avoidance response (Figure 2A, left).

#### Auxin Transport

In the hypocotyl, the auxin efflux carrier genes *PIN3* (*PIN-*FORMED3) and *PIN7* are rapidly induced by low R:FR treatment, and induced by low blue light (LBL) treatment (Devlin et al., 2003; Keuskamp et al., 2010, 2011; Kohnen et al., 2016). Consistent with potential functions of PIN3 and PIN7 in hypocotyl elongation, low R:FR and LBL-induced hypocotyl elongation is significantly reduced in the *pin3 pin4 pin7* triple mutant. This indicates that polar auxin transport from the cotyledon to the hypocotyl is required for the shade avoidance response (Keuskamp et al., 2010; Kohnen et al., 2016).

The low induction of *PIN* genes compared to the extremely high induction of other auxin-related genes (such as *YUC8, IAA19*, and *IAA29*) suggests that the rapid response of PIN-type transporters to shade signals might not primarily occur through transcriptional regulation. Consistent with other levels of regulation participating in this response, the protein abundance and subcellular localization of PIN3 are rapidly regulated by low R:FR shade signal (Keuskamp et al., 2010). *D6 PROTEIN KINASE* (*D6PK*) and its homolog *D6PK-LIKE1* (*D6PKL1*), encode two shade-induced protein kinases that function in the phosphorylation of PINs. Disruption of *D6PK* and *D6PKL1* leads to inhibited elongation under low R:FR conditions, indicating that the phosphorylation of PINs is required for the shade response (Figure 2A, middle; Keuskamp et al., 2010; Barbosa et al., 2014; Kohnen et al., 2016).

Although PIN3, PIN4, and PIN7 are known to be involved in the shade response, how various shade signals regulate their transcription and subcellular localization, thus leading to the rapid transport of auxin from the cotyledon to the hypocotyl, remains to be explored in the future. In addition to PINs, ATP-binding cassette B (ABCB) auxin carriers also play a significant role in the shade avoidance response (Ge et al., 2017).

#### Auxin Signaling

*SMALL AUXIN UP-REGULATED RNA* (*SAUR*) genes are the largest family of early auxin-response genes (Ren and Gray, 2015). *SAUR9, 10, 19, 20, 22*, and *23* are rapidly induced by shade treatment, suggesting they might be involved in the shade avoidance response (Devlin et al., 2003; Kohnen et al., 2016). Overexpressing *SAUR19* in the *pif4* mutant background completely rescued the *pif4* phenotype of impaired hypocotyl elongation at high temperatures and overexpressing *SAUR36* also promoted hypocotyl elongation (Franklin et al., 2011; Stamm and Kumar, 2013). However, the mechanisms by which these SAURs mediate shade-induced cell elongation remain to be demonstrated. Interestingly, recent studies have shown that SAUR19-mediated elongation might involve the direct activation of plasma membrane H^+^-ATPase, leading to cell wall acidification and loosening, thereby facilitating cell expansion (Spartz et al., 2014, 2017; Ren and Gray, 2015; Fendrych et al., 2016). These findings suggest that SAUR proteins could mediate shade-promoted cell elongation by regulating cell wall acidification and loosening (Figure 2A, middle).

AUXIN RESPONSE FACTOR-type transcription factors and Auxin/Indole Acetic Acid (Aux/IAA)-type repressors are transcriptional regulators that form homo- and hetero-oligomers and play crucial roles in the transcriptional regulation of auxin response genes. The *arf6 arf7 arf8* triple mutant fails to respond to low R:FR treatment, indicating that ARF6, ARF7, and ARF8 are required for shade-induce cell elongation (Reed et al., 2018). Although many *Aux/IAA* genes (e.g., *IAA1, IAA5, IAA19, IAA29*, and *IAA30*) are rapidly and strongly induced by various shade signals, how they mediate shade responses remains unclear (Kohnen et al., 2016). A recent study suggested that IAA19 and IAA29 mediate shade-induced cell elongation possibly by inhibiting the transcription of *IAA17* (Pucciariello et al., 2018). Consistent with this, *IAA17* is highly expressed in the hypocotyl epidermal cells and the gain-of-function mutant *iaa17-1* showed impaired shade-induced hypocotyl elongation, indicating that IAA17 inhibits the shade avoidance response (Procko et al., 2016). In addition, IAA19 physically interacts with ARF7 mediating auxin- and brassinosteroid (BR)-regulated phototropic responses of hypocotyls (Harper et al., 2000; Tatematsu et al., 2004; Zhou et al., 2013). Therefore, in addition to the auxin pathway, IAA19-mediated shade responses might involve the BR signal pathway (Zhou et al., 2013).

### Auxin Sensitivity Increases Under Prolonged Shade Conditions

The expression of auxin biosynthetic genes in cotyledons, the lamina, and petioles is rapidly induced within hours after plants perceive a low R:FR shade signal (de Wit et al., 2015). However, auxin contents rapidly decrease to basal levels after 24 h of low R:FR treatment, suggesting that auxin-mediated prolonged shade responses might not involve the regulation of auxin biosynthesis (de Wit et al., 2015). The response to prolonged shade conditions requires a system-wide rearrangement of auxin perception and signaling transduction (Pucciariello et al., 2018). Under these conditions, reduced phyB activity leads to decreased PIF4 levels in cotyledon mesophyll cells, and increased levels in hypocotyl vascular cells, which promotes *IAA19* and *IAA29* expression. IAA19 and IAA29 then suppress the expression of *IAA17*, thus promoting growth. Meanwhile, the transcript levels of the genes encoding the auxin receptors TRANSPORT INHIBITOR RESPONSE1 (TIR1) and AUXIN SIGNALING F-BOX (AFB) proteins are significantly increased, thus enhancing auxin perception and signal transduction under prolonged shade conditions (Pucciariello et al., 2018).

After prolonged low R:FR shade treatment, lamina width decreases but petiole length increases. This contrast in growth might be caused by differences in auxin sensitivity, because the auxin contents do not dramatically differ between the lamina and petiole (Figure 1B; de Wit et al., 2015). PIF7 is required for this contrast growth, but how PIF7 differentially modifies auxin sensitivity in the petiole vs. lamina, with contrasting effects on growth, remains poor understood (Figure 2A, right; de Wit et al., 2015, 2018).

Under prolonged shade conditions, free IAA and BR decrease to basal levels after approximately 24 h, while gibberellic acid (GA) contents continue to increase, suggesting that GA might play an important role in the response to prolonged shade (Bou-Torrent et al., 2014). In addition, PIF4 and PIF5 directly suppress the expression of *MIR156*; miR156 further reduces the transcript abundance of its targets *SQUAMOSA-PROMOTER BINDING PROTEIN-LIKE* (*SPL*) family genes, thus mediating the prolonged shade response (Figure 2A, right; Xie et al., 2017; Wei et al., 2018).

It is worth noting that decreased photosynthetic activity leads to reduced sucrose contents under prolonged shade conditions; this might contribute to the morphological and physiological changes in these plants. Indeed, reducing the expression of *SUCROSE TRANSPORTER 4* (*SUT4*) significantly inhibited the shade avoidance response in potato (Kozuka et al., 2005; Chincinska et al., 2008). Consistent with a role of sugar signaling in shade responses, sugar transport in the phloem and local starch metabolism are required for shade-induced cell elongation of Arabidopsis hypocotyls (de Wit et al., 2018).

### Response to Low Blue Light (LBL) Shade Signals

Arabidopsis PIF4 and PIF5 are not only involved in low R:FR responses, but also participate in LBL responses (Keller et al., 2011; de Wit et al., 2016; Goyal et al., 2016; Pedmale et al., 2016). LBL strongly induces the expression of *PIF4* and *PIF5* in the apical portion of the hypocotyl and increases the stability of PIF5 (Sun et al., 2013; Pedmale et al., 2016). After LBL treatment, *pif4* and *pif5* seedling plants show significantly reduced hypocotyl and petiole elongation, and reduced hyponastic leaf growth. These observations indicate that PIF4 and PIF5 are essential for the LBL-induced shade response, with PIF4 playing a more important role (Keller et al., 2011; de Wit et al., 2016; Pedmale et al., 2016).

Although PIF4 and PIF5 are involved in both low R:FR- and LBL-induced shade responses, the underlying mechanisms are distinct. PIF4 and PIF5 mediate the low R:FR response by regulating the expression of auxin biosynthesis, transport and signaling-related genes (Figure 2A, middle; Hornitschek et al., 2012). By contrast, they mediate the LBL response mainly by regulating the expression of genes encoding cell wall-modifying enzymes (Pedmale et al., 2016). Although free IAA levels do not rapidly increase after LBL treatment, the LBL response is significantly reduced in *taa1, pin3 pin4 pin7*, and *tir1 afb1 afb2 afb3* seedlings, indicating that plant responses to LBL require auxin synthesis, transport, and signaling transduction (Keuskamp et al., 2011; Pedmale et al., 2016). In addition, *pif7* mutant seedlings showed a partial response to LBL treatment, indicating that PIF7 is also required for LBL responses (Pedmale et al., 2016).

### Interaction of Photoreceptors and PIFs During Shade Responses

High R:FR increases the ratio of the active form of phyB (Pfr, the far-red light-absorbing form) in the nucleus; Pfr physically interacts with PIFs and promotes their phosphorylation, ubiquitination, and degradation (Leivar and Quail, 2011; Leivar and Monte, 2014). Low R:FR increases the ratio of the inactive form of phyB (red light-absorbing form, Pr) in the cytoplasm, thus increasing PIF accumulation in the nucleus and promoting the expression of their downstream target genes (Figure 2B; Casal, 2013). Consequently, PIF3, PIF4, and PIF5 but not PIF7 are rapidly degraded under high R:FR conditions, but their levels rapidly increase after low R:FR shade treatment (Lorrain et al., 2008; Leivar et al., 2012b).

PIF4 and PIF5 directly bind to the promoters of auxin biosynthetic genes *YUC8, TAA1*, and *CYP79B2* and auxin-responsive genes *IAA19, IAA29*, and *SAUR19-24* and activate their expression in response to shade, as well as phototropic and high-temperature responses (Franklin et al., 2011; Sun et al., 2013; Ma et al., 2016). Under low PAR but high R:FR conditions, PIF4 and PIF5 also bind to the promoters of auxin biosynthesis and signaling genes, suggesting that low PAR-induced cell elongation is also dependent on PIF4- and PIF5-mediated auxin responses (Hornitschek et al., 2012).

In contrast to the effect of low R:FR on PIF4 and PIF5 stability and accumulation, low R:FR induces PIF7 de-phosphorylation and translocation from the cytoplasm to the nucleus, thus enhancing PIF7 binding to the promoters of auxin-related genes such as, *YUC8, YUC9, IAA19*, and *GH3.3* (Hornitschek et al., 2012; Li et al., 2012; Huang et al., 2018). In turn, PIF7 interacts with H3K4me3 and H3K36me3-READER MOTIF RELATED GENE2 (MRG2) and recruits it to the coding regions of various targets of PIF7 further regulate their expression (Figure 2B; Huang et al., 2018; Peng et al., 2018). Consistent with this, disrupting both *MRG1* and *MRG2* resulted in a reduced shade response, indicating that they positively regulate the shade response (Peng et al., 2018).

High-intensity red and blue light promotes PIF4 and PIF5 protein phosphorylation and degradation, thus inhibiting the transcription of growth-promoting genes and suppressing hypocotyl elongation (Figure 2A, left). Under LBL, photoreceptors cryptochrome-1 (CRY1) and CRY2 physically interact with PIF4 and PIF5. In turn, CRY2 is recruited to the promoters of PIF4 and PIF5 targets and modulates their expression (Figure 2B; Pedmale et al., 2016). By contrast, under high blue light, CRY1 physically interacts with PIF4 and represses its effects on transcription, thus suppressing hypocotyl elongation under high temperature conditions (Ma et al., 2016). In addition, the UV-B photoreceptor UVR8 negatively regulates low R:FR shade and high temperature induced auxin biosynthesis and cell elongation partially by enhancing the degradation or inhibited the transcriptional activity of PIF4 on its targets (Hayes et al., 2014, 2017). Therefore, PIF proteins physically interact with multiple different kinds of photoreceptors and mediate cell elongation through different regulatory mechanisms.

### Auxin and Light Antagonistically Regulate the Stability of Aux/IAA Proteins

In general, under low free IAA conditions, Aux/IAA repressors physically interact with ARF transcription factors and inhibit their function, thus negatively regulating auxin responses. Under high free IAA conditions, Aux/IAA proteins are rapidly degraded, thus releasing its repression on ARFs, which in turn promotes the expression of ARFs downstream targets and induces auxin responses (Leyser, 2018). Interestingly, the stability of Aux/IAA proteins is also controlled by light (Huq, 2018; Xu et al., 2018; Yang et al., 2018). In the dark, phyB and CRY1 are present in the cytoplasm in inactive forms that cannot physically interact with Aux/IAA proteins. Under high-intensity red- or blue-light, phyB or CRY1 interact with Aux/IAA proteins in a light-dependent manner, thus inhibiting their protein degradation and decreasing hypocotyl elongation (Xu et al., 2018; Figure 2). Under deep shade (very low R:FR) conditions, phyA protein stability significantly increases. In turn, phyA physically interacts with Aux/IAA proteins and prevents their degradation, thus negatively regulating shade avoidance responses (Yang et al., 2018). These studies revealed that Aux/IAA stability is rapidly regulated by auxin receptors and photoreceptors, which allow plants to perceive endogenous auxin content and environmental light signaling thus to fine-tune cell elongation under different light conditions.

## Understanding Shade Responses: Future Perspectives

In the past decades, the mechanisms regulating the shade responses have been well studied in the model plant Arabidopsis, however, it will take much longer to transfer this knowledge to crop plants. Our recent study revealed that ectopic expression of maize *PIF4* and *PIF5* in Arabidopsis *pifq* mutant completely rescued its impaired shade avoidance response, which indicated that PIF proteins might play conserved roles in shade response (Shi et al., 2018b). Transcriptome analyses further indicated that maize and Arabidopsis might share very conserved regulatory pathways of shade avoidance response (Wang et al., 2016; Shi et al., 2018a). Besides the well-known shade avoidance response, how shade-tolerant plants increase their survival and fitness in various shade conditions remains to be further investigated. A recent study suggested that the biosynthesis and signaling of auxin, GA, and BR are not significantly affected by shade signals in the shade-tolerant *Geranium robertianum*, suggesting that differing patterns of hormones might be one of the reasons for the different morphogenic and physiological changes in shade tolerant vs. intolerant species (Gommers et al., 2018). Interestingly, the clonal plant *Potentilla reptans* uses different strategies to respond to various shade signals, including enhanced vertical growth in the presence of (simulated) short-dense neighbors, shade tolerance behavior in the presence of tall-dense neighbors, and lateral avoidance behavior in the presence of tall-sparse neighbors (Gruntman et al., 2017). Indeed, both vertical growth and lateral avoidance behaviors are phototropisms or types of directional growth, consistent with the finding that plants tend to reposition their growth towards unfiltered sunlight at the edges or gaps of natural canopies (de Wit et al., 2016; Goyal et al., 2016; Fiorucci and Fankhauser, 2017). These findings provide new perspectives about shade responses in the natural environment.

## Author Contributions

Both authors conceived the study, and wrote and revised the manuscript.

## Conflict of Interest Statement

The authors declare that the research was conducted in the absence of any commercial or financial relationships that could be construed as a potential conflict of interest.
